# Superficial siderosis of the central nervous system: A case report

**DOI:** 10.3892/etm.2015.2229

**Published:** 2015-01-28

**Authors:** JI-GUO GAO, CHUN-KUI ZHOU, JING-YAO LIU

**Affiliations:** Department of Neurology, First Hospital of Jilin University, Changchun, Jilin 130031, P.R. China

**Keywords:** superficial siderosis, hearing loss, magnetic resonance imaging

## Abstract

Superficial siderosis of the central nervous system (SSCNS) is a rare syndrome resulting from hemosiderin deposits in neuronal tissues close to the cerebrospinal fluid. SSCNS is characterized by sensorineural deafness, cerebellar ataxia and signs of pyramidal tract dysfunction. The present study describes a patient with SSCNS that did not suffer from hearing loss, which is the most common symptom of SSCNS. The patient was a 48-year-old male, presenting with dizziness, ataxia and slurred speech. The patient’s ataxia was characterized by dizziness, nystagmus, dysarthria, abnormal finger-nose pointing and heel-knee-shin tests and a positive Chaddock sign. The patient had suffered from a pontine hemorrhage two years prior to the study. Audiometric tests showed normal hearing during the hospital stay and at the two-month follow-up examination. The diagnosis of SSCNS was made based on magnetic resonance images, which showed areas of linear hypointensity on the surface of the pons with mild cerebellar atrophy. However, a long-term follow-up is required to monitor the hearing of the patient. Improved understanding of SSCNS is important for clinicians to identify SSCNS patients who present without typical clinical symptoms.

## Introduction

Superficial siderosis of the central nervous system (SSCNS) is a rare disease of the CNS resulting from hemosiderin deposits in neuronal tissues close to the cerebrospinal fluid. Since this disease was first reported by Hamill in 1908 (1), there have been only ~300 reported cases of SSCNS in the literature (2). The clinical syndrome is characterized by sensorineural deafness, cerebellar ataxia, dementia and signs of pyramidal tract dysfunction (3). SSCNS is mainly caused by subarachnoid hemorrhage due to idiopathic bleeding, which accounts for 35% of all cases (4). Other known causes of subarachnoid hemorrhage leading to SSCNS include brain tumors, trauma and vascular abnormalities (4). To date, no effective treatment is available for SSCNS. In the past, SSCNS was primarily diagnosed by postmortem examination or surgical biopsy (3). However, with advances in radiological imaging techniques, cranial magnetic resonance imaging (MRI) has become the primary method for the diagnosis of SSCNS (5). MRI can detect the presence of hemosiderin on the surface of the brain and spinal cord with considerable sensitivity.

The present study reports the case of a 48-year-old male patient who was diagnosed with SSCNS using MRI. The patient presented with dizziness, ataxia and nystagmus, but without the typical SSCNS symptom of hearing loss.

## Case report

A 48-year-old male patient was admitted to the First Hospital of Jilin University (Changchun, China) on October 11^th^ 2013 after presenting with dizziness, ataxia and slurred speech, with symptoms aggravating over five months. The patient’s speech had begun to slur five months previously with no evident cause; however, the patient did not suffer from any communication difficulties. The patient also exhibited ataxia and had lost the ability to walk independently; however, the lower extremities of the patient moved normally while in the supine position. These symptoms worsened progressively, developing into blurred vision and dizziness. The patient exhibited no hearing loss in either ear, and no headache, nausea or vomiting. The patient had suffered from a pontine hemorrhage two years previously, with a sequelae of mild slurred speech and ataxic gait, but was able to walk independently at that time. In addition, the patient had experienced hypertension for two years, but had no history of diabetes or venereal disease exposure. Written informed consent was obtained from the patient prior to their inclusion in the present study.

On examination, the patient was conscious and exhibited dysarthria. Both eyes exhibited horizontal and rotary nystagmus, and the pupils were round and equal in diameter (3.0 mm) with a normal pupillary light reflex. The patient showed normal symmetrical nasolabial folds, a normal midline tongue protrusion, bilateral powerful lift of the soft palate and a reduced gag reflex. Muscle strength was 5/5 in the extremities, with normal muscle tone and tendon reflexes. The Chaddock sign was positive, and the deep and shallow sensations were normal. Responses to the finger-nose pointing and heel-knee-shin tests were unsteady and inaccurate. The patient exhibited a positive Romberg’s sign, while meningeal irritation and the Kernig sign were negative.

Laboratory tests were normal, consisting of routine blood and urine analyses, blood coagulation, liver and kidney functions and blood ion concentrations. In addition, the patient’s antinuclear antibody, anti-O antibody, erythrocyte sedimentation rate, thyroid function and tumor markers were all normal. A lumbar puncture revealed clear and colorless cerebrospinal fluid with a pressure of 160 mmH_2_O. The white blood cell count was zero, the protein level was 0.31 g/l, the glucose level was 2.9 mmol/l and the chloride content was 126 mmol/l in the cerebrospinal fluid. Pandy’s test, as well as tests for *Mycobacterium tuberculosis*, *Cryptococcus neoformans*, *Treponema pallidum* and HIV were all negative.

**Figure 1:**
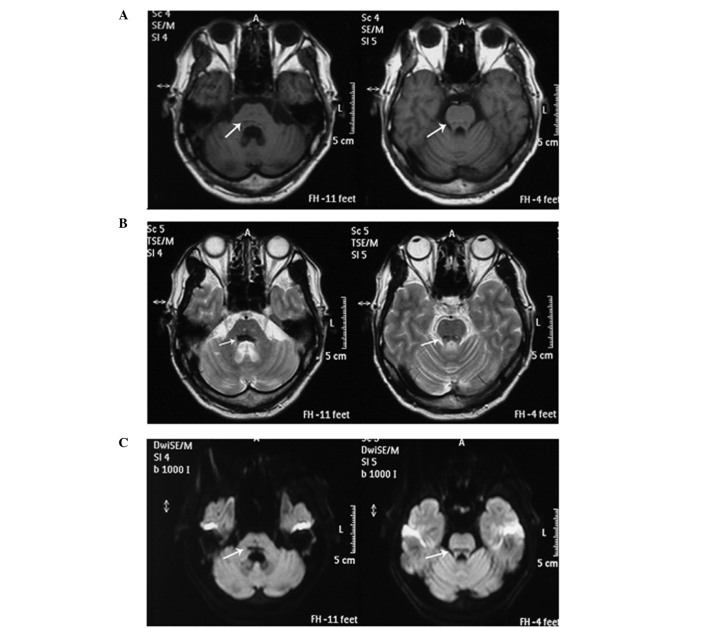
Cranial magnetic resonance images showing areas of linear hypointensity (arrows) on the surface of the pons, using (A) T1-weighted imaging (T1WI), (B) T2-weighted imaging (T2WI) and (C) diffusion-weighted imaging (DWI). Images revealed widened and deepened sulci and gyri, an enlarged ambient cistern and mild cerebellar atrophy. The size of hypointensity due to hemosiderin was larger in the T2WI when compared with the T1WI and DWI.

Cross-sectional MRI scans of the cranium were captured using T1-weighted imaging (T1WI), T2-weighted imaging (T2WI) and diffusion-weighted imaging (DWI), which revealed areas of linear hypointensity on the surface of the pons (Fig. 1). The size of hypointensity due to hemosiderin was larger in the T2WI scans than in the T1WI and DWI scans. The images revealed widened and deepened sulci and gyri, an enlarged ambient cistern and mild cerebellar atrophy. The patient received oral administration of vitamins B1 (20 mg, 3 times/day) and B12 (0.05 mg, 3 times/day) for 2 months. The patient was followed up at 2 months following discharge. Audiometric tests showed normal hearing during the hospital stay and at the two-month follow-up examination.

## Discussion

SSCNS is a rare disease caused by chronic repeated subarachnoid hemorrhage (3). Clinical symptoms can occur as late as 8–37 years after the initial onset of the subarachnoid hemorrhage (6). In the present study, the patient showed a slow disease progression, presenting with clinical symptoms of ataxia, dizziness and slurred speech two years after the initial pontine hemorrhage.

Idiopathic bleeding is the major source of subarachnoid bleeding, accounting for 35% of all cases, which is followed by CNS tumors (15%), brain trauma (13%) and arteriovenous malformation (9%) (4). Other rare sources of subarachnoid bleeding include subdural surgery, brachial plexus injury and nerve root avulsion. The pathology of SSCNS primarily results from hemosiderin deposits and subsequent impairment of the surface of the cerebrum, cerebellar vermis, cranial nerve VIII and corpora quadrigemina, as well as the longitudinal and lateral fissures and sulci of the brain (7) and pia mater of the spinal cord (8). Macroscopically, a brownish discoloration is observed in the leptomeninges and the adjacent surface of the brain parenchyma and ventricular walls. Adhesion in the subarachnoid space is often observed. Microscopically, there is hemosiderin deposition, leptomeningeal fibrosis, neuronal loss and increased macrophage infiltration, as well as reactive gliosis, axonal demyelination and intracellular ovoid bodies (9).

Prior to the development of MRI, the diagnosis of SSCNS depended primarily on postmortem examination or surgical biopsy (3). MRI has become the primary method for diagnosis of SSCNS since first being used for this purpose in 1985 (5). The appearance of SSCNS in MRI scans is due to the presence of ferric ions on the surface of CNS tissues (5). Compared with T2WI, head susceptibility-weighted imaging is more sensitive to hemosiderin and can more accurately detect hypointense signals in the brain (3), which provides more valuable imaging information for the diagnosis of SSCNS. Although fast fluid-attenuated inversion-recovery MRI is sensitive to acute subarachnoid hemorrhage (10), it is limited in the detection of hemosiderin deposits.

T2WI MRI reveals a characteristic linear hypointensity due to hemosiderin deposition that is in contrast to the hyperintense signals of the cerebrospinal fluid (11). Therefore, since the characteristic hypointensity is easily detected by T2WI MRI, this technique is often used to diagnose SSCNS. In addition, cerebellar atrophy is commonly identified by cranial MRI (12). In the present case, a characteristic linear hypointensity on the surface of the pons with mild cerebellar atrophy was observed in the T2WI MRI scans, supporting the diagnosis of SSCNS.

The characteristic clinical features of SSCNS are bilateral sensorineural deafness, progressive cerebellar ataxia and pyramidal tract signs (3). Ali *et al* (13) reported that sensorineural deafness occurred in 95% of SSCNS cases, ataxia in 88% and signs of pyramidal tract dysfunction in 76%. In cases of ataxia due to injury of the cerebellar vermis, gait ataxia occurs more commonly than limb ataxia in SSCNS patients (8). Other clinical symptoms of SSCNS include dementia, loss of smell, bladder dysfunction, somatic sensory dysfunction, unequally sized pupils, headache and back pain (14). In addition, certain SSCNS patients experience the sudden onset of headaches and meningitis-like symptoms (15). In rare cases, extraocular muscle paralysis, dysarthria, sciatica and motor neuron damage in the lower extremities also occur. In the early stages of the disease, symmetrical or asymmetrical high-frequency hearing loss often occurs, and dizziness can occur when the brain lesions involve cranial nerve VIII (3).

In the present case, the 48-year-old patient presented with clinical symptoms and signs of ataxia that consisted of dizziness, nystagmus, dysarthria and abnormal finger-nose pointing and heel-knee-shin tests two years after suffering from a pontine hemorrhage. In addition, the patient exhibited a positive Chaddock sign, suggesting that the pyramidal tract was impaired. These clinical symptoms were consistent with the diagnosis of SSCNS. The characteristic areas of linear hypointensity on MRI further confirmed the diagnosis of SSCNS.

In the present case, SSCNS was likely to have been caused by the pontine hemorrhage. The onset of clinical symptoms varies greatly among SSCNS patients. A number of SSCNS patients present no clinical symptoms during their lifetime and are only diagnosed by postmortem autopsy. In rare cases, SSCNS patients may present clinical symptoms in the early stages of the disease; however, the majority of patients exhibit no clinical symptoms (16). SSCNS may be found incidentally by cranial MRI, showing the presence of hemosiderin deposits. The onset and duration of clinical symptoms may be determined by the source and extent of the bleeding.

One of the most common clinical symptoms of SSCNS is sensorineural deafness. However, in the present case, the patient did not present with any signs of hearing loss. The development of sensorineural deafness is a chronic process and the progressive deterioration of hearing commonly lasts for several years until hearing is completely lost (17). Although the current patient had normal hearing at the two-month follow-up examination, a long-term follow-up is required to monitor the hearing of the patient.

Currently, no effective treatment is available for SSCNS. Surgery is feasible for the removal of blood from the site of hemorrhage, and it is possible to surgically correct the cause of the subarachnoid hemorrhage, such as nerve root avulsion, arteriovenous malformation and dural lesions. However, surgery has been effective in very few patients, with a number of patients experiencing aggravated symptoms following surgery (18). Ion chelation, large doses of vitamin C and E and hormones (alone or in combination with immunosuppressive therapy) have been clinically used, and are able to effectively halt disease progression in certain patients (19). Han *et al* (20) found that penicillamine was effective in the treatment of SSCNS. In the present case, hormone therapy with dexamethasone (started at a 15-mg dose and gradually tapered, once daily for one month) was found to reduce dizziness, with the patient able to walk with the aid of a walker after receiving the treatment.

In summary, SSCNS is a rare disease that is easily overlooked by clinicians. The present study described the case of an SSCNS patient who presented with dizziness, ataxia and nystagmus, without loss of hearing. MRI is important for the diagnosis of SSCNS, and improving the understanding of SSCNS is important for clinicians to identify SSCNS patients who do not exhibit typical clinical symptoms.
